# Effects of widespread community use of face masks on communication, participation, and quality of life in Australia during the COVID-19 pandemic

**DOI:** 10.1186/s41235-022-00436-z

**Published:** 2022-10-01

**Authors:** Karyn L. Galvin, Dani Tomlin, Lynette Joubert, Lauren Story

**Affiliations:** 1 Department of Audiology and Speech Pathology, The University of Melbourne, 550 Swanston St, Parkville, Melbourne, VIC 3053 Australia; 2 Department of Social Work, The University of Melbourne, Melbourne, Australia

## Abstract

The objective was to document the influence of face mask use by other people on communication experiences, participation in activities, and quality of life. Australian adults (*n* = 665) completed an online survey; 90.8% resided in a state with mandatory mask use outside the home and 44.1% self-reported hearing difficulties. Mask use was reported as negatively affecting communication quality in the community (90.2%) and workplace (91.8%), and with household members (59.1%), including an increased requirement for clarification and repetition, increased difficulty communicating, and decreased understanding. Masks influenced feelings when communicating in the community (74.1%) and workplace (76.7%), and with household members (43.6%), including increased fatigue and frustration, and decreased connection to others. Masks influenced the time spent communicating in the community (68.8%) and workplace (67.9%), and with household members (42.3%), including a decrease in the number of individuals communicated with, and the time spent communicating with each individual. Masks influenced participation in activities in the community (50.9%) and workplace (59.7%), and with household members (41.3%), including reduced participation in health-related activities, shopping, and socialising. Influences on quality of life included reduced physical and mental health, including increased loneliness. Female gender and greater self-reported hearing difficulties were significantly associated with increased influence of mask use. The wide-ranging influences of face mask use have implications for physical health and mental health, including social connectedness, and for employers and the economy. As an important measure for combatting disease spread, the negative impacts of mask use must be considered during policy formulation, and appropriate mitigating measures, such as educational campaigns, enacted.

**Supplementary Information:**

The online version contains supplementary material available at 10.1186/s41235-022-00436-z.

## Introduction

To curb the spread of disease during the COVID-19 pandemic, face masks have been worn in many countries since 2020, either voluntarily or in response to public health regulations. Covering the face limits access to visual cues which are important during communication, such as facial expressions and lip movements (Mheidly et al., 2020; Summerfield, 1992). The use of face masks has been shown to result in significant communication challenges for deaf and hard-of-hearing listeners, with the acoustic signal compromised by spectral changes, including reduced transmission of high frequency information (above 1 kHz) and attenuation of the signal (Atcherson et al., 2017; Corey et al., 2020; Magee et al., 2020; Palmiero et al., 2016). While the use of transparent masks allows better access to visual cues, these were found to create even greater acoustic deficits than non-transparent masks, with more resonant peaks, sound attenuation and deflection occurring (Atcherson et al., 2021). Even for listeners with normal hearing, reduced quality of life due to facemasks has been demonstrated due to the attenuation of sounds and loss of facial expressions creating communication difficulties (Malzanni et al., 2021).

Theoretical and anecdotal reports discussing the impact of face mask use in the community have been presented on traditional and social media platforms, and in letters and editorials (see, for example, Chodosh et al., 2020; Graydon et al., 2020; Munro, 2020). Much of this material focussed on people living with hearing impairment, given that these individuals are likely to be particularly impacted by the decreased access to visual and acoustic cues due to the use of face masks during communication, with less attention given to the experiences of the broader community. In a letter-to-the-editor, preliminary data collected from 59 individuals with hearing impairment who attended an Italian emergency room in February 2020 indicated that 86% experienced some difficulty related to their hearing impairment, and that their main concerns about face masks related to sound attenuation (44%) and lack of lipreading cues (56%) (Trecca et al., 2020). In a more extensive report of a larger study, survey results were presented regarding the effects of various ‘lockdown’ measures on 129 people living with hearing impairment in Glasgow (Naylor et al., 2020). At the time (early June 2020), restrictions were being eased after 2 months of limited local travel, mandatory face mask wearing during health consultations, and social distancing. Responses to items related to the impact of face mask use indicated that participants found it harder to understand people due to the loss of visual cues (74%) and “muffled” speech (89%), were worried about how they would communicate if mask wearing became more common (54%), and were anxious about going to public places where they may need to speak to people wearing face masks (46%). Only for the final item was there a difference between a better hearing (33%) and a worse hearing group (60%), indicating that those with worse hearing had more difficulty following COVID-19 updates on the radio. The wider implications of the communication difficulties experienced by participants were not explored in this rapid research study which examined multiple other aspects of life under lockdown, including increased use of video-conferencing and reduced access to hearing care. There is still limited empirical evidence of the type and extent of impact from face mask use experienced both by those with hearing impairment and the broader community (Oosthuizen et al., 2022).

A large online survey of 460 UK residents in mid-2020 by Saunders et al. (2021) was undertaken which included periods of mandatory mask use in various settings, such as shops and public transport. The investigators purposely oversampled adults with hearing impairment and asked questions about perceptions of face coverings from a public health perspective and communication while wearing a face covering. In addition, the survey asked questions about communicating with other people who were wearing face coverings in specific situations, such as a doctor’s appointment, or communicating on public transport. Participants generally reported that face coverings negatively impacted hearing, understanding, engagement and connection. Qualitative content analysis of open-ended responses identified themes which included interpersonal changes during communication, negative impacts on the interaction (such as reduced informal chatting), and negative impacts on the individual (such as feeling anxious, stressed, isolated, frustrated, and fatigued). Most participants (83%) provided open-ended content, and the authors considered this to reflect the high levels of concern about the impact of face coverings. Thus, there is some evidence that the use of face coverings impacted more than just communication ease. The authors noted that widespread face mask use was not mandatory during the entire data collection period, and that it was important for future, communication-related research to examine the impact of widespread use of face masks.

The radical public health measures which have been put in place across the globe (such as face mask use, physical distancing, work-from-home mandates, closure of public gathering spaces) were necessary to combat the spread of COVID-19. Some of these measures are likely to be required for some time as new variants emerge and vaccination rates vary across countries. Furthermore, the global experience during the pandemic is likely to influence future public health responses and, indeed, voluntary uptake of measures which reduce disease transmission. The world may well see more consistent use of face masks in certain higher risk contexts, such as healthcare settings and public transport, and even future periods of widespread community use of face masks. To facilitate informed decision making by governments regarding public health regulations in the future, it is important that the harmful consequences of public health interventions against COVID-19 are considered (Bavli et al., 2020). To achieve this, it is first necessary to have a clear understanding of the impact of public health interventions, such as the mandatory use of face masks. This impact should be considered not only for those assumed to be more at risk (for example, individuals who are deaf or hard-of-hearing), but also for the broader population. Inclusion of the broader population in previous studies has been limited, as these studies have focussed only on, or intentionally oversampled, individuals who have hearing impairment. Moreover, impact should be considered not only for the specific behaviour most likely to be impacted (in this case communication ease and engagement/connection during communication), but also more broadly. Other effects on communication, such as decreased time spent communicating with other people, and secondary effects, such as restricted activity participation, are possible and are likely to compound negative effects already occurring in crisis situations such as pandemics. From an economic point of view, it is also important to consider effects on communication in the workplace, which has not been addressed in previous studies. The aim of this study was to understand the impact of very widespread community use of face masks on communication, activity participation, and quality of life for adults in the general community in a range of common communication settings. The first objective was to document the influence of face mask use by other people on communication experiences when communicating with community members in general, with household members specifically, and in the workplace. The second objective was to document the secondary effects of widespread use of face masks by other people on participation in activities in these same settings, and on quality of life.

## Methods and materials

### Participants

Ethical approval for the study was obtained from the University of Melbourne’s Office of Research Ethics and Integrity. Participants were members of the general public residing in Australia who were 18 years or older. Although all Australian residents were eligible, advertising to recruit participants was primarily circulated within the state of Victoria, where face mask use was mandatory (refer to Procedure section below). As the survey was completed anonymously, no incentive or feedback was provided to participants.

### Survey

Data were collected using an online survey accessed via the Qualtrics platform (https://www.qualtrics.com/au/). The authors (three audiologists (KG, DT, LS) and one social worker and psychologist (LJ)) constructed the survey items, and revised them based on feedback on clarity, ease of reading, and adequate coverage of the relevant issues provided by 3 audiologists, 1 speech pathologist, and 2 community members who piloted the first draft. Full details of the final survey items and response options are provided as Additional file 1: Digital Content 1. In the survey, participants were asked if face mask use by other people influenced the quality of their communication, their feelings related to communication, the activities they participated in, and the time they spent communicating in three different communication settings. The response options were: No, not at all influential, Somewhat influential, and Very influential. The settings were: communicating with community members in the community, with people in the workplace, and with household members when away from their home environment. Survey branching ensured that only participants who indicated that they had communicated with other people wearing face masks in a particular setting were presented with items related to that setting. Participants were also asked if face mask use by other people influenced their quality of life; quality of life in general was examined as the authors considered that it would be too difficult to connect aspects of quality-of-life (such as sleep quality and happiness) to specific communication settings. Participants who indicated some degree of influence were subsequently presented with additional items. These additional items were most often specific questions regarding the type and direction of influence; for example, participants indicating influence on time spent communicating were subsequently presented with items asking if they communicated with more people or fewer people, and if they communicated for more time or less time per person. Where participants had indicated influence on participation in activities, these additional items were open-set questions asking which activities were engaged in more, or less, due to other people wearing face masks. For the community setting only, the additional items also included a list of suggested activities which may have been engaged in more, or less (for example, shopping for non-essential items, attending non-medical health-care appointments).

Additional questions focused on activities and behaviours which could improve communication, with these results to be reported separately.

### Procedure

Participants were recruited via snowball sampling: the link to the relevant Qualtrics webpage was circulated via email and social media to the authors’ personal and professional networks with the aim to advertise as widely as possible to potential participants with and without hearing difficulties. Participants who accessed the online data collection survey via the link were initially presented with a participant information form and provided their consent via a tick box at the end of the form which then allowed them access to the survey items. Data were collected from October to early December 2020. In the state of Victoria, wearing a face mask was mandatory in all settings outside the home from August 2nd 2020 and throughout the data collection period (Victorian Government Department of Health & Human Services, 2020). Face mask use was encouraged in other Australians states but was not consistently mandatory in all settings as case numbers were low, and so the recruitment effort primarily targeted Victorian residents. It is important to note that, at the time, Australia had a far lower infection rate (total cases 28,000; December 10th 2020) ((Victorian Government Department of Health & Human Services, 2020) and a low number of deaths due to COVID-19 (908; December 10th 2020) (Victorian Government Department of Health & Human Services, 2020) compared with many other countries. Even in Victoria, the state with the most cases by far at the time, there were only 195 active cases of COVID-19 amongst the population of 6.7 million when data collection commenced on October 1st, and there were no new cases of COVID-19 after October 30th. There were zero active cases by November 29th, and no new active cases through to the end of data collection. Thus, although mask wearing was mandatory in Victoria during this time, the impact of the pandemic was relatively low compared with most other countries.

There were also other COVID-19-related measures in place during the data collection period, which varied across locations and loosened with time. During the data collection period of October 1st to early December, Victorian residents were in some form of “lockdown” from October 1st to 27th. Even where and when lockdown restrictions were harshest (in the capital city of Melbourne from October 1st to 18th), residents were allowed to leave their home every day for exercise, limited outdoor socialising, essential shopping, healthcare, and permitted or essential work; single-person households were allowed a visitor; and small weddings, funerals and religious gatherings could be held. By early November, relatively few non-mask related restrictions remained, although people who could perform their work from home were still required to work from home. Other states outside Victoria had generally lower-level restrictions or none.

### Analysis

The survey data were downloaded from the Qualtrics platform on December 17, 2020. The data were then imported into the Minitab software for statistical analysis. Descriptive statistics were calculated to describe the demographic characteristics of the participant group divided into those self-reporting pre-existing hearing difficulties and those reporting none. Descriptive statistics (percentage reporting face mask use by other people was not influential, somewhat influential, or very influential) were calculated for communication quality, feelings related to communication, time spent communicating, and participation in activities in the three different communication settings, and on overall quality of life. McNemar’s tests were used to compare the proportion of participants reporting no influence of face mask use by other people versus the proportion reporting some influence (i.e. with the categories of somewhat influential and very influential combined) across the different communication settings. The tests were repeated for each aspect of communication (quality, feelings, and time) and for participation in activities. For those participants who reported influence of face mask use by other people, additional McNemar’s tests were used to compare, across communication settings, the proportion of participants who reported a worsened outcome (e.g. more difficulty communicating) versus the proportion who reported a similar or improved outcome. For these McNemar’s tests, a Bonferroni adjusted alpha level of 0.0167 was utilised to account for the multiple comparisons required to compare each setting to the other. The sample sizes varied as the McNemar’s test utilises paired data, so that only those participants who answered items related to the settings being compared could be included in a particular test.

Ordinal logistic regression analyses were conducted on the response data to consider the factors which may have been associated with the degree of influence reported for face mask use by other people. A regression analysis was conducted for each aspect of communication (quality, feelings, and time) and for participation in activities for each of the three communication settings, as well as for quality of life. The factors of gender and degree of self-reported hearing difficulties were entered into the initial model; non-significant factors were removed (as detailed in Table 2) and the analysis repeated. The reference levels and their comparators were male (compared to female) and no self-reported hearing difficulties (compared to mild, moderate, significant, and very significant difficulties). Only the gender categories of female and male were included as other levels of the gender factor contained small numbers of participants, namely gender variant/non-conforming (*n* = 4), transgender male (*n* = 1), and unspecified (*n* = 3). Age was not included as a factor due to it being correlated with degree of hearing difficulties. A significant positive coefficient for the level of a particular factor would be interpreted as meaning that participants were more likely than those in the reference level to provide a response at the top of the response list, or closer to the top. Very influential was the response at the top of the list, and Somewhat influential was second (i.e. closer to the top than Not influential).

For those participants who reported that face mask use by other people influenced their quality of life, additional McNemar’s tests were used to compare, across the aspects of quality of life, the proportion of participants who reported a worsened outcome (e.g. poorer sleep) versus the proportion who reported a similar or improved outcome. For these McNemar’s tests, a Bonferroni adjusted alpha level of 0.008 was utilised to account for the multiple comparisons required.

## Results

Demographic information for the 665 participants who completed the survey is presented in Table 1. Of the 665 participants, 293 self-reported experiencing hearing difficulties prior to the onset of the COVID-19 pandemic. The distribution of ages was representative of the population, matching the 2016 Victorian population to within 3% for each decade of age, i.e. 21–30, 31–40, 41–50, etc. (Australian Bureau of Statistics, 2020). The male to female ratio was approximately 1–5. The majority (90.8%) of participants provided a Victorian postcode (the state with mandatory mask use and where advertising was targeted).

**Table 1:** Summary of participant characteristics for the 665 participants divided into groups with and without self-reported pre-existing hearing difficulties

Demographic factor	Level	Number (%) of participants	
		Hearing difficulties (*n* = 293)	No hearing difficulties (*n* = 372)
Age	18–40	68 (23.2%)	188 (50.5%)
	41–60	94 (32.1%)	142 (38.2%)
	> 60	131 (44.7%)	42 (11.3%)
Gender	Male	56 (19.1%)	51 (13.7%)
	Female	232 (79.2%)	318 (85.5%)
	Non-conforming	4 (1.4%)	0 (0.0%)
	Transgender male	0 (0.0%)	1 (0.3%)
	Unspecified	1 (0.3%)	2 (0.5%)
Degree of self-reported hearing difficulties	None	N/A	372 (100%)
	Mild^1^	153 (52.2%)	N/A
	Moderate^2^	77 (26.3%)	N/A
	Significant^3^	33 (11.3%)	N/A
	Very significant^4^	30 (10.2%)	N/A

Examples of performance provided for participants to self-report degree of hearing difficulties: ^1^Some difficulty understanding conversation in background noise. ^2^Need TV louder than others; difficulty understanding conversation in background noise. ^3^Cannot follow a group conversation in a quiet room. ^4^Cannot understand one person speaking in a quiet room if I can’t see their face

As noted above, items related to a particular setting were only presented to participants who reported communicating in that setting with other people wearing face masks. Of the 665 participants, 644 reported leaving home and communicating with people wearing face masks in the community. These participants reported leaving home a maximum of 2 times per week (14%), 3–5 times per week (40.8%), 1–2 times per day (35.3%), or more than twice per day (9.9%). They reported communicating with people wearing face masks in the community a maximum of 1 time on each occasion they left home (13%), 2–4 times on each occasion they left home (45.2%), 5–10 times on each occasion they left home (24.4%), or more than 10 times on each occasion they left home (17.4%). Of the 665 participants, 377 reported working in a workplace in which they communicated with people wearing face masks. These participants reported working 1–4 days per fortnight (40.1%), 5–7 days per fortnight (26.5%) or 8–10 days per fortnight (33.4%). They reported communicating with people wearing face masks in the workplace a maximum of 1 time per day (4.5%), 2–4 times per day (10.6%), 5–10 times per day (14.6%), or more than 10 times per day (70.3%). Of the 665 participants, 477 reported communicating with household members wearing face masks when away from the home environment. Of these 477 participants, 56.4% lived with at least one child, and 43.7% lived only with other adults. They reported communicating with household members wearing face masks a maximum of 2 times per week (30.2%), 3–5 times per week (45%), 1–2 times per day (20%) or more than twice daily (5.0%).

### Influence on aspects of communication, on participation in activities, and on quality-of-life

**Fig. 1:**
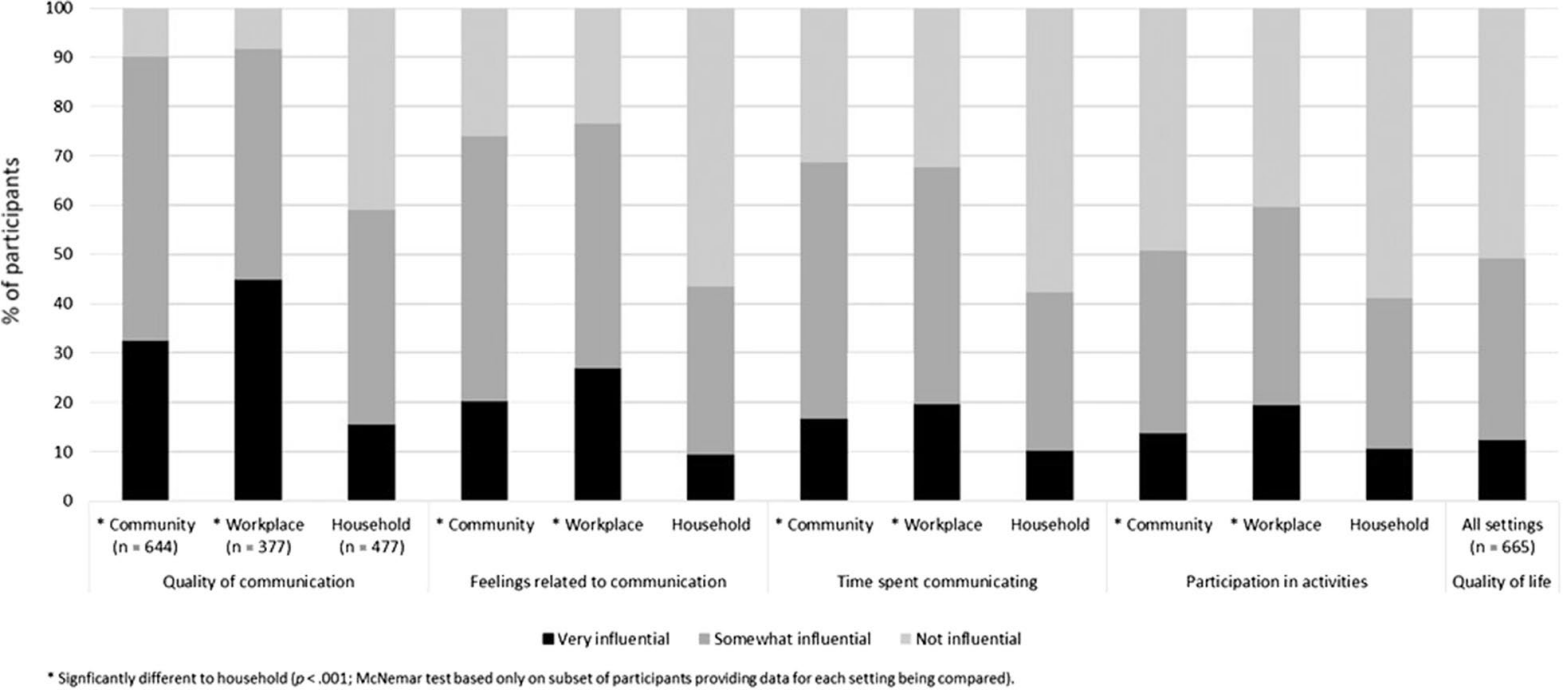
Percentage of participants who reported face mask use by other people was very, somewhat, or not influential on different aspects of communication and participation in activities when communicating in the community (*n* = 644), in the workplace (*n* = 377), and with household members (*n* = 477), and on quality of life in general (*n* = 665)

Figure 1 presents the percentage of participants who reported that face mask use by other people was very influential, somewhat influential, or not influential on three aspects of communication (quality of communication, feelings related to communication, and time spent communicating), on participation in activities, and on overall quality of life. Data for all variables except quality-of-life are presented for each of the three communication settings. The percentage of participants reporting that face mask use by other people was somewhat influential or very influential on the three aspects of communication (quality, feelings, and time) in the settings of the community and the workplace was high (67.9–91.8%). Quality of communication was particularly affected, with 90.2% and 91.8% of participants, respectively, reporting some degree of influence of face mask use by other people in the community and the workplace settings. McNemar’s tests showed that the proportion of participants reporting that face mask use by other people was somewhat influential or very influential was significantly higher for communicating in the community (*n* = 475; *p* < 0.001) and in the workplace (*n* = 288; *p* < 0.001) compared to with household members, across all three aspects of communication (quality, feelings, and time). Although lower than for other communication settings, the percentage of participants reporting influence of face mask use by other people on communication with household members was still substantial (42.3–59.1%) across the three aspects of communication. McNemar’s tests showed that the percentage of participants reporting influence of face mask use by other people on participation in activities was also significantly higher for communicating in the community (*n* = 475; *p* < 0.001) and in the workplace (*n* = 288; *p* < 0.001) compared to with household members. Half of the participants reported that face mask use by other people influenced their quality of life.

Table 2 provides a summary of the outcomes of the ordinal logistic regression analyses, indicating the levels of the factors (gender and degree of self-reported hearing difficulties) that were significantly associated with the reported influence of face mask use by other people on the individual outcome variables. This is in comparison with the reference levels of each factor (i.e. male gender and no self-reported hearing difficulties). As shown, female gender and greater degree of self-reported hearing difficulties were significantly associated with being more likely to provide a response at the top of the list, or closer to the top (i.e. to report that face mask use by other people was very influential, or somewhat influential) for a number of aspects of communication across the three settings, as well as on participation in activities and on quality of life. More specifically, self-reported significant or very significant hearing difficulty was significantly associated with being more likely to report that face mask use was very influential, or somewhat influential, on all aspects of communication (quality, feelings, and time) in the community, on participation in activities in the community, and on quality of life. The same was true for self-reported moderate hearing difficulties, with the exception of the association with time spent communicating and participation in activities. In the analysis of data related to communication in the workplace, self-reported degree of hearing difficulty was not a significant predictive variable and was removed from the model. In the analysis of data related to communication with household members, significant or very significant self-reported hearing difficulties were also significantly associated with being more likely to report that face mask use was very influential, or somewhat influential, on the quality of communication, feelings related to communication (very significant difficulties only), and time spent communicating (significant difficulties only). Female gender was significantly associated with being more likely to report that face mask use by other people was very influential, or somewhat influential, on all aspects of communication across all settings, and on quality of life. In general, the odds ratios are larger for the significant and very significant degrees of self-reported hearing difficulties, indicating that these predictors have a larger impact on the response variable (i.e. influence) than did moderate hearing difficulties or female gender. For example, participants who self-reported very significant hearing difficulties were 19.35 times more likely than those who self-reported no hearing difficulties to report that face mask use by other people was very influential or somewhat influential on communication quality when communicating in the community. As a further illustration, Table 3 provides the data on reported influence of face mask use by other people on communication quality when communicating in the community as a function of gender and as a function of degree of self-reported hearing difficulties.

**Table 2:** Summarised outcomes of ordinal logistic regression analyses: factor levels with a significant effect on reported greater or lesser influence on the outcome variables for communication in the community, in the workplace or with household members

Setting/variable	Factor (reference level)											
	Gender (male)			Self-reported hearing difficulties (No hearing difficulties)								
	Female			Moderate			Significant			Very significant		
	*p*	Coeff	OR	*p*	Coeff	OR	*p*	Coeff	OR	*p*	Coeff	OR
*In the community*												
Quality	< .001	1.13	3.10	< .001	1.02	2.77	< .001	1.840	6.31	< .001	2.85	17.35
Feelings	.013	0.52	1.68	.005	0.70	3.29	< .001	1.81	12.55	< .001	2.23	21.05
Time	Non-significant factor; removed from model						.006	0.97	2.65	.001	1.31	3.69
Participation							.001	1.17	3.21	< .001	1.83	6.24
*In the workplace*												
Quality	.017	0.70	2.01	Non-significant factor; removed from model								
Feelings	.011	0.72	2.06	Non-significant factor; removed from model								
*With household members*												
Quality	.024	0.53	1.70				.004	1.31	3.69	< .001	1.87	6.52
Feelings	.021	0.59	1.80							.020	1.04	2.83
Time	.021	0.60	1.82				.011	1.14	3.12			
Participation	.018	0.62	1.85	Non-significant factor; removed from model								
*No specific setting*												
Qual of Life	0.032	0.45	1.57	.032	0.51	1.67	.018	0.81	2.25	.004	1.02	2.76

**Table 3:** Example data for the reported influence of face mask use as a function of gender and degree of self-reported hearing difficulties

Factor/level	Influence on communication quality in the community		
	Not influential	Somewhat influential	Very influential
*Gender*			
Male	22.1	54.8	23.1
Female	6.7	58.4	34.9
*Degree of hearing difficulties*			
None	10.31	61.84	27.86
Mild	11.03	68.28	20.69
Moderate	4.05	48.65	47.30
Very significant	3.70	11.11	85.19
Significant	6.25	25.00	68.75

### Type and direction of influence on aspects of communication

**Fig. 2:**
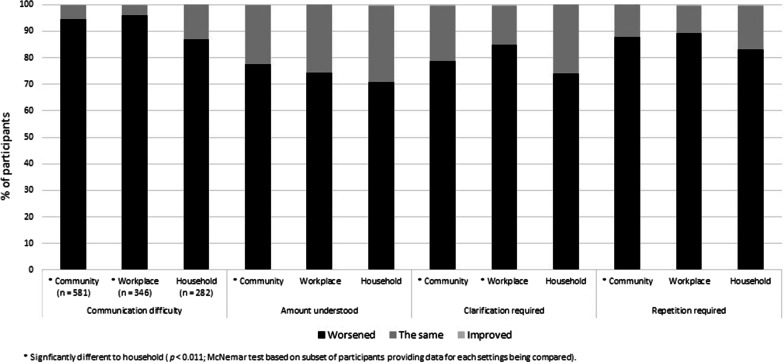
Percentage of participants reporting face mask use by other people was very or somewhat influential on communication quality who reported a worsened, the same, or improved impact on four measures of communication quality when communicating in the community (*n* = 581), in the workplace (*n* = 346), and with household members (*n* = 282)

**Fig. 3:**
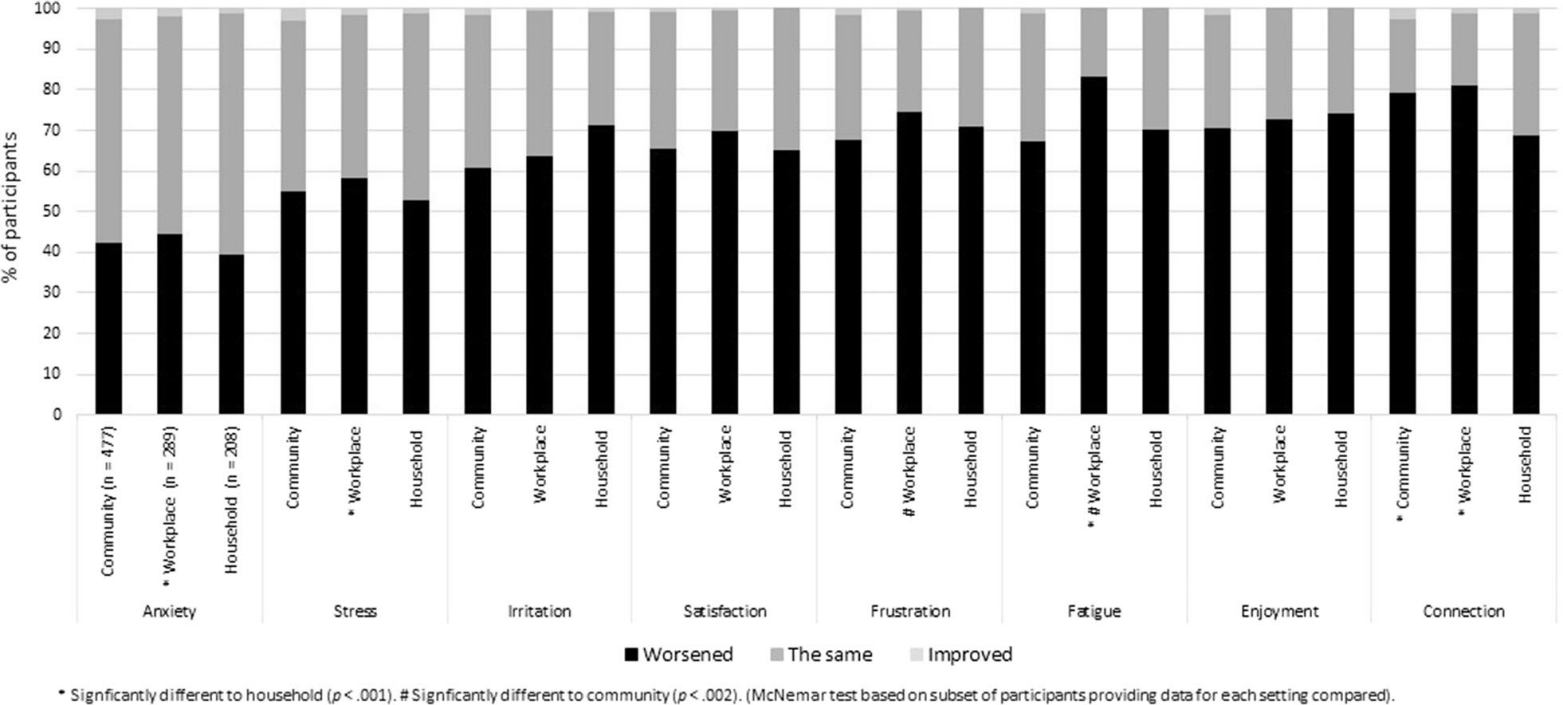
Percentage of participants reporting face mask use by other people was very or somewhat influential on feelings related to communication who reported a worsened, the same, or improved impact on eight feelings when communicating in the community (*n* = 447), in the workplace (*n* = 289), and with household members (*n* = 208)

**Fig. 4:**
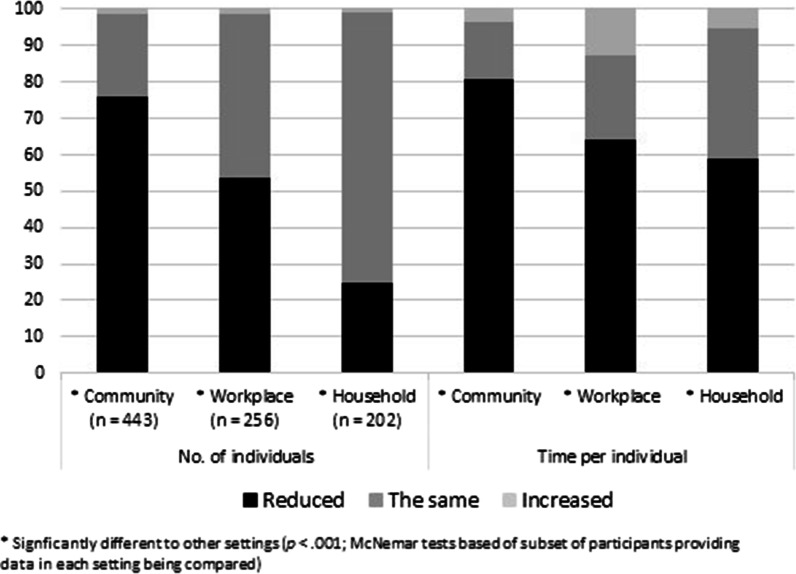
Percentage of participants reporting face mask use by other people was very or somewhat influential on time spent communicating who reported a worsened, the same, or improved impact on the number of individuals communicated with and the time spent communicating per individual in the community (*n* = 443), in the workplace (*n* = 256), and with household members (*n* = 202)

Data relating to the type and direction of influence of face mask use by other people on communication quality, feelings related to communication, and time spent communicating in each of the three communication settings are presented in Figs. 2, 3, and 4, respectively. As shown in Fig. 2, for the four aspects of communication quality (communication difficulty, amount understood, clarification required, and repetition required) between 70.9 and 96% of participants reporting influence of face mask use by other people reported a worsened outcome. With regard to the proportion of participants reporting a worsened outcome, versus the same or an improved outcome, for each aspect of communication, there were some significant differences across communication settings. McNemar’s tests indicated that a worsened outcome (i.e. decreased amount understood, and increased communication difficulty, and requirement for clarification and repetition) was reported by a significantly greater proportion of participants for communication in the community compared to communication with household members (*n* = 163, *p* ≤ 0.004). Moreover, increased communication difficulty and requirement for clarification was reported by a significantly greater proportion of participants for communication in the workplace compared to communication with household members (*n* = 166, *p* ≤ 0.011). There were no significant differences between the community and workplace settings (*n* = 249, *p* ≥ 0.066).

As shown in Fig. 3, of those participants who reported influence of face mask use by other people on feelings related to communication, between 39.4 and 83.4% reported worsened levels of anxiety, stress, irritation, satisfaction, fatigue, frustration, enjoyment, and connection to others. These feelings are presented in Fig. 3 in generally increasing percentage, with frustration, fatigue, enjoyment, and connection to others being the feelings with the highest percentage of participants reporting worsened feelings. McNemar’s tests indicated that increased feelings of anxiety, stress, and fatigue, and decreased connection to others, were reported by a significantly greater proportion of participants for communication in the workplace compared to communication with household members (*n* = 119, *p* < 0.002). Moreover, increased fatigue and frustration was reported by a significantly greater proportion of participants for communication in the workplace compared to communication in the community (*n* = 182, *p* ≤ 0.002). Finally, decreased connection to others was reported by a significantly greater proportion of participants for communication in the community compared to communication with household members (*n* = 113, *p* < 0.001).

### Type and direction of influence on time spent communicating

As shown in Fig. 4, of those participants who reported influence of face mask use by other people on time spent communicating, at least half reported a decrease in the number of individuals they communicated with and the time spent per individual across the three communication settings, with one exception: only 25.2% of participants reported a reduction in the number of individuals communicated with when communicating with household members. Comparisons across settings using McNemar’s test indicated that a decrease in the number of individuals communicated with, and the time spent communicating per individual, was reported by a significantly higher proportion of participants for communication in the community compared to each of communication in the workplace (*n* = 173, *p* < 0.001) and communication with household members (*n* = 107, *p* < 0.001). Moreover, a decrease in the number of individuals communicated with, and the time spent communicating per individual, was reported by a significantly higher proportion of participants for communication in the workplace compared to communication with members household (*n* = 103, *p* < 0.001).

### Type and direction of influence on activity participation

Of the 328 participants (50.9% of the total participants) who reported influence of face mask use by other people on participation in activities when communicating in the community, 27.8–36% reported less participation in important activities such as shopping for essential items and/or attending medical and health appointments (refer to Table 4). A higher percentage of participants reported influence of face mask use by other people on less essential activities, with 47.9–73% of the 328 participants reporting less participation in non-health appointments, visiting cafes and restaurants, shopping, and/or socialising. In response to the open-set question regarding “other activities”, participants reported less ‘non-verbal communication’ and less ‘community training/volunteer work’.

**Table 4:** Impact of face mask use by other people on participation in specific activities in the community and the reported influence on participation (*n* = 328)

Activity	Reported influence on participation			
	N/A^1^	More	The same	Less
Shop for medicines (*n* = 327^2^)	11.3	3.4	69.1	16.2
Shop for essential items (*n* = 327)	2.1	3.7	66.4	27.8
Medical appointments (*n* = 327)	6.4	2.1	61.2	30.3
Health appointments (*n* = 325)	14.5	2.8	46.8	36.0
Take away food (*n* = 327)	7.6	11.3	41.6	39.4
Accessing services^3^ (*n* = 327)	7.6	1.8	49.8	40.7
Exercising (*n* = 327)	2.4	8.0	45.9	43.7
Non-health appointments (*n* = 326)	11.3	3.1	37.7	47.9
Café/restaurant (*n* = 327)	11.6	3.7	26.9	57.8
Non-essential shopping (n = 327)	7.0	3.7	30.9	58.4
Planned socialising (*n* = 326)	4.6	2.8	33.1	59.5
Incidental socialising (*n* = 326)	4.0	3.7	19.3	73.0

^1^Not applicable; either activity not performed by the individual or not allowed under COVID-19 restrictions. ^2^Sample size varies as not all 328 participants who reported an influence on activities provided a response indicating the degree of influence for each activity. ^3^For example, bank or post office

The 59.7% of participants who reported influence of face mask use by other people on participation in activities in the workplace were presented with open-set questions requesting them to identify the activities impacted. Activities participants reported engaging in less were communicating with colleagues, customers, clients, and patients; formal and informal meetings with colleagues; and team/relationship building. Activities participants reported engaging in more were online interactions, including videoconferencing and telehealth, although participants did not specify if these were mandated activities.

The 41.3% of participants who reported influence of face mask use by other people on participation in activities with household members when away from their home environment were also presented with open-set questions requesting them to identify the activities impacted. Activities participants reported engaging in more included activities to improve communication (such as seeking clarification). Activities participants reported engaging in less included “going out together” (as it was too hard to communicate), exercise, shopping, communicating, and social outings such as having meals in cafes.

### Type and direction of influence on quality-of-life

**Fig. 5:**
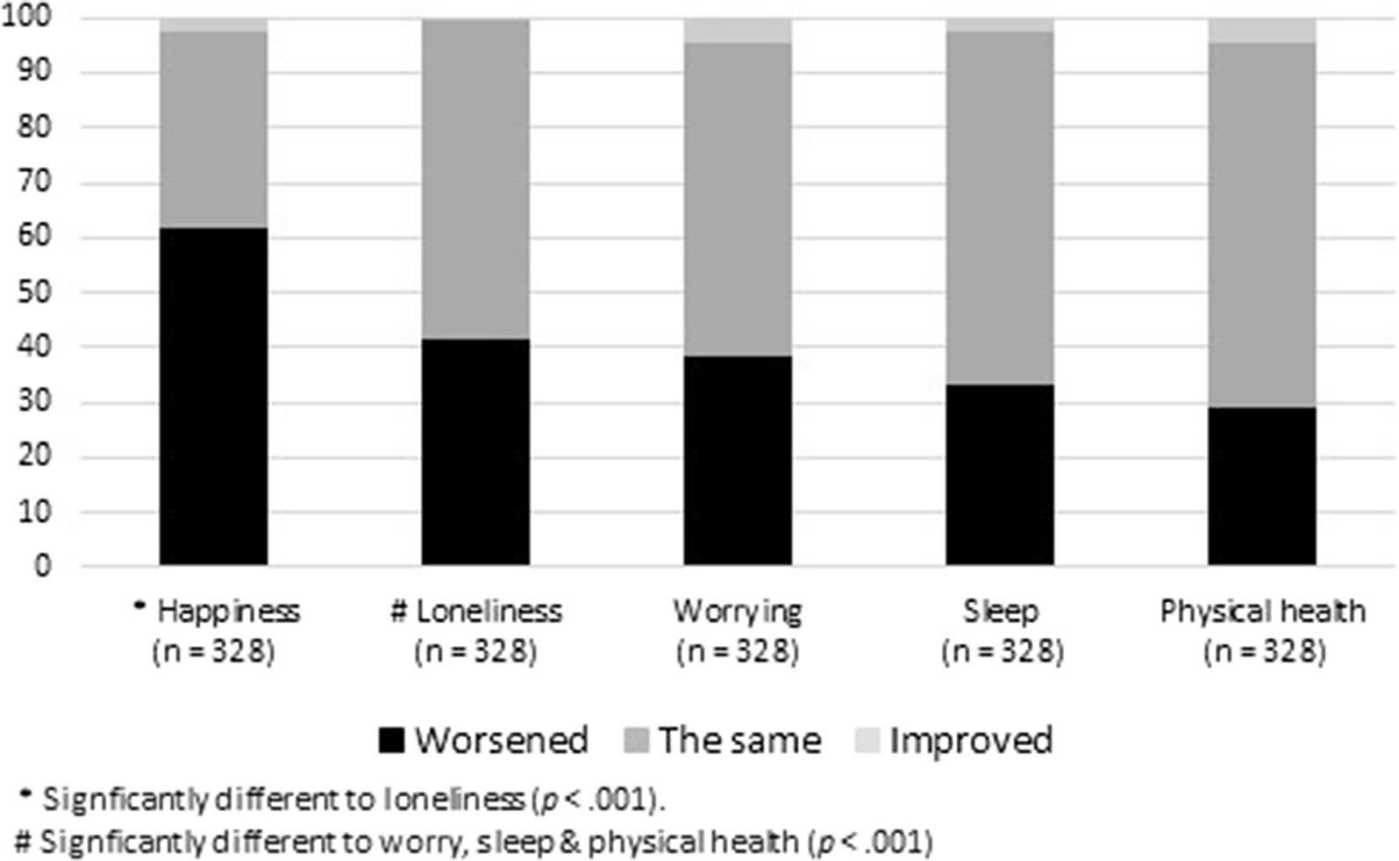
Percentage of participants reporting face mask use by other people was very or somewhat influential on their quality of life (*n* = 328) who reported a worsened, the same, or improved impact on five aspects of quality of life

The 49.3% of participants who reported influence of face mask use by other people on their quality-of-life were presented with additional questions related to specific aspects of quality of life. As shown in Fig. 5, around one-third of the participants reporting influence, reported poorer physical health and sleep, and increased levels of worrying. McNemar’s tests indicated that the proportion of participants reporting increased levels of loneliness was significantly greater than the proportion reporting increased levels of worrying, poorer physical health, or poorer sleep (*p* < 0.001). The proportion of participants reporting decreased levels of happiness was significantly greater than the proportion reporting higher levels of loneliness (*p* < 0.001).

### Illustrative findings for the total group

**Fig. 6:**
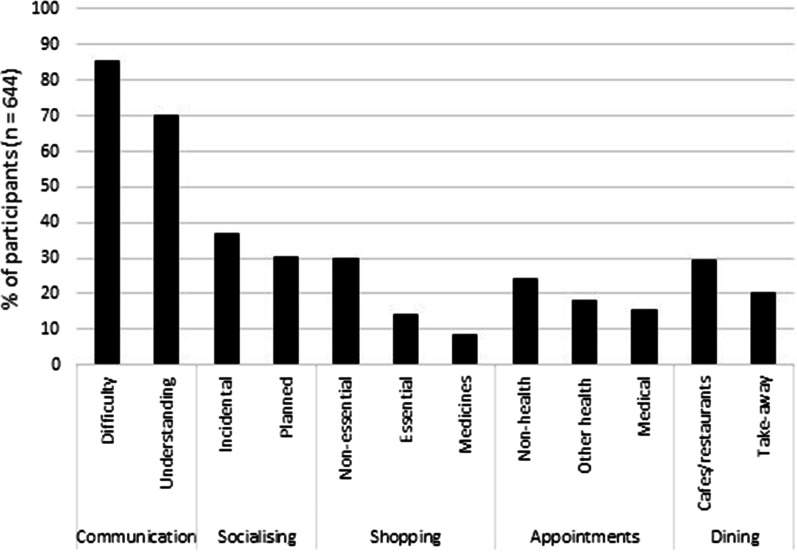
Percentage of the total group of participants who reported communicating with other people wearing face masks in the community (*n* = 644) who reported a worsened outcome in selected aspects of communication, and decreased participation in selected activities

**Fig. 7:**
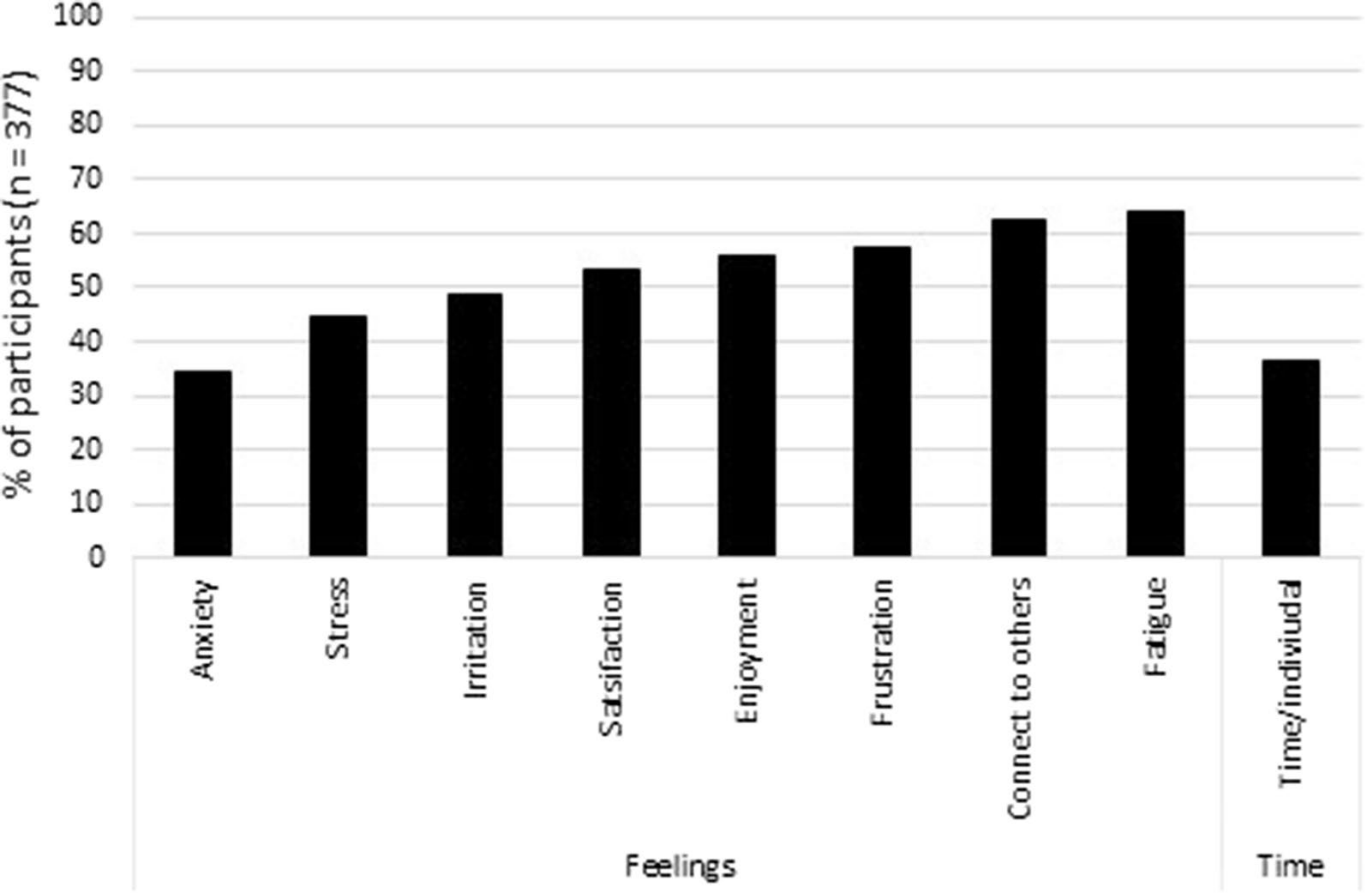
Percentage of the total group of participants who reported communicating with other people wearing face masks in the workplace (*n* = 377) who reported a worsened outcome on feelings related to communication, and decreased time spent communicating

Some illustrative findings regarding communication and activity participation in the community are presented in Fig. 6. The percentage of participants who reported a worsened outcome for each variable is expressed as a percentage of the overall group who reported communicating with people wearing face masks in the community (i.e. 644 participants), rather than as a percentage of the group who reported influence (as is the case in Figs. 2 and 3). Some illustrative findings regarding feelings related to communication and time spent communicating in the workplace are presented in Fig. 7. The percentage of participants who reported a worsened outcome for each variable is expressed as a percentage of the overall group who reported communicating with people wearing face masks in the workplace (i.e. 377 participants), rather than as a percentage of the group who reported influence (as is the case in Figs. 3 and 4).

## Discussion

Results from this survey indicated that use of face masks by other people had multiple detrimental impacts on communication across different settings. This study has shown that for a high proportion of survey participants, face mask use by other people influenced the quality of their communication, their feelings about their communication, the number of individuals they communicated with, and the time they spent communicating per individual (Figs. 1 and 4). These influences were reported when communicating with community members in the community, in the workplace, and with household members when away from their home environment. Moreover, these effects on communication experiences led to changes in activity participation and, for half of the participants, impacted their quality-of-life (Fig. 1 and Table 4).

The influence of face mask use by other people on communication was evident and widespread across the various aspects of communication and different communication partners. Nearly all participants (~ 90%) reported an influence on the quality of their communication in the community (Fig. 1). As shown in Fig. 2, of the 581 participants reporting influence of face masks in the community, 94.7% reported more communication difficulty and 77.6% reported they understood less. This represents 85.4% and 70.0%, respectively, of the total group of 644 who reported communicating with people in the community wearing face masks (refer to Fig. 6). These percentages are consistent with the approximately 75% of participants who reported the ability to understand when face coverings were worn was harder or much harder for specific situations experienced by between 75 and 247 people (Saunders et al., 2021). Not surprisingly, there were negative secondary effects for the participants in the present study, including how they felt about communication in the community, the time they spent communicating, and the activities they participated in. These results are consistent with the themes identified in the study by Saunders et al. (2021), in which participants responded to open-ended questions, reporting an array of negative feelings and impact on social connectedness. Given the importance of social connection to physical and mental health (Holt-Lunstad, 2021), the findings related to reduced time spent communicating are crucial. Also important from a social connection point of view is the influence of face mask use by other people on participation in social activities. Approximately one-third of the total group of 644 participants who engaged in communication in the community reported reduced participation in each of planned (30.1%) and incidental (37.0%) socialising due to face mask use by other people (refer to Fig. 6).

For participants with significant or very significant self-reported hearing difficulties, the use of face masks by other people was significantly more likely to have a greater influence on their quality of life, on all aspects of their communication and on participation in activities in the community, and on some aspects of their communication with household members. For participants with moderate self-reported hearing difficulties, the use of face masks by other people was significantly more likely to have a greater influence on their quality of life, and on the quality of communication and their feelings related to communication in the community (refer to Table 2). The increased likelihood was in comparison with participants with no self-reported hearing difficulties. In each case, the odds ratios indicate that the increase in likelihood was greatest for those with very significant self-reported hearing difficulties (ORs 2.76–21.05), followed by significant difficulties (ORs 2.25–12.55), and then moderate difficulties (ORs 1.67–3.29) (refer to Table 2). There were no such significant results for self-reported mild hearing difficulties, indicating similar experiences when communicating with others wearing face masks for participants with mild self-reported hearing difficulties and no hearing difficulties (refer to Table 3 for an example). There were also no significant associations for communication in the workplace. It is noteworthy that for the workplace analysis, the sample sizes for the categories self-reported significant or very significant hearing difficulties were small, at 8 and 8, respectively, and this may have affected power. The associations with degree of hearing difficulties are not unexpected. Saunders et al. (2021) also found that increased degree of self-reported hearing loss was associated with finding it harder to hear, and experiencing decreased engagement, when conversing with family and friends as well as in medical appointments when others were wearing face coverings. Given the vulnerability of individuals experiencing hearing difficulties, who already report high levels of listening and communication effort (Alhanbali et al., 2017; Beechey et al., 2020), communication breakdown and miscommunication (Cudmore et al., 2017; Stevens et al., 2019), the importance of any additional communication burden cannot be underestimated. The present results show that even a moderate degree of self-reported difficulties is associated with around triple the likelihood of reporting greater influence on both quality of communication (OR 2.77, *p* < 0.001) and feelings related to communication (OR 3.29,* p* < 0.001) in the community. Moreover, they show that a significant degree of self-reported hearing difficulties is associated with almost triple the likelihood of reporting greater influence on time spent communicating in the community (OR 2.65, *p* = 0.006) and with household members (OR 3.12, *p* = 0.011). These are also particularly concerning findings, given the associations between hearing loss, loneliness, and social isolation for adults (Bott & Saunders, 2021; Shukla et al., 2020) and between hearing loss and mental health impacts, such as higher levels of depression (Adigun, 2017).

For female participants, the use of face masks by other people was more likely to have a greater influence on quality of life, the quality of communication and feelings related to communication across all three setting, and on the time spent communicating and participation in activities with household members. It is possible that women reported more influence than men due to typical gender differences in communication styles, in the time devoted to verbal communication, and in the value placed on verbal communication and its use as a tool to build social relationships (Merchant, 2012). There is already significant research demonstrating greater impact of the COVID-19 pandemic for women in terms of domestic burden (Sevilla & Smith, 2020), paid employment, and physical and mental health (Nordhues et al., 2021). If, as appears to be the case here, women in Australia experienced more negative influences due to face mask use by other people, then this compounds the additional impacts already documented, and perhaps limits the opportunities for women to mitigate other effects through their usual social interactions.

The influence of face mask use by other people resulting in decreased participation in activities in the community is relevant to health in general and to the economy. A decrease in participation was reported by a small but substantial percentage of the total group engaged in communication in the community across a range of health-related activities, including attending medical (15.4%) and non-medical appointments (18.2%), and shopping for medicines (8.2%) and essential items (14.1%) (refer to Fig. 6). These percentages of participants reporting decreased participation in health-related activities are lower than the percentages of participants reporting decreased participation in other non-health related activities (such as non-health appointments; 24.0%). Nevertheless, there was a decrease in participation in health-related activities, and it is important to consider the consequences of this. Decreased participation in health-related activities may have a minor impact on health (e.g. temporary reduction in consumption of fresh fruit) through to a major impact (e.g. a missed or delayed cancer diagnosis due to not attending screening appointments). There is emerging evidence of such outcomes during the pandemic, for example decreased consumption of fruit and vegetables (Sidor & Rzymski, 2020), decreased or delayed diagnosis of cancer (Maringe et al., 2020), and longer delays between symptom onset and hospital treatment for cardiovascular disease (Kiss et al., 2021). The inclusion of specific situations in their online survey allowed Saunders et al. (2021) to conclude that more people were impacted by face mask use in medical situations than during more informal communication. The qualitative data reported by Saunders et al. (2021) also indicated that accompanying persons were very often not allowed to attend appointments due to the infection risk, so that patients had to rely only on their own ability to understand the health practitioner wearing a face mask. The difficulty of doing this may well have contributed to the reduction in health-related activities documented in the present study.

An economic impact can be anticipated when face mask use reduces participation in activities which involve financial outlay. These include not only some health-related activities as described above, but also other activities. Of the total group of 644 engaged in communication with community members, up to 30% reported decreased participation in eating at cafes and restaurants (29.3%), non-essential shopping (29.7%), non-health related appointments (24.2%), and purchasing take-away food (20%) (refer to Fig. 6). Decreased participation at such a high rate across a range of activities would reduce turnover for many business types and have secondary effects across the local economy where face mask use is widespread or mandated. It is noteworthy that there were also small increases in participation in some activities, due perhaps to a shift towards those activities requiring less face-to-face communication as, for example, a shift from restaurant to take-away meals.

During the data collection period, there were COVID-19 related restrictions on workplaces, especially in Victoria, where residents were encouraged or required (when possible) to work from home at different time points. Nevertheless, 56.7% of participants (*n* = 377) reported communicating with other people wearing face masks in a workplace, and 33.4% of these reported working outside the home almost full time. Of these 377, 91.8% reported influence of face mask use on communication quality (Fig. 1), such that the majority found communication more difficult, required more repetition and clarification, and understood less. More difficult and less successful communication is likely to impact at an individual and organisational level. Workers need to communicate with supervisors and colleagues to carry out their assigned duties safely and successfully. They also need to communicate with suppliers and clients to fulfil their individual role and to contribute to achieving the overall aims of operating the business or organisation. Moreover, workplace communication in a social context is important for the development of a connected and supportive environment which facilitates worker engagement and retention. For the total group of 377 participants who reported communicating in the workplace, the difficulty of communication when others were wearing face masks increased negative feelings of anxiety (34.2%), stress (44.8%), irritation (48.8%), and frustration (57.3%), and decreased positive feelings of satisfaction (53.6%) and enjoyment (56.0%) (refer to Fig. 7). Particularly important was the percentage reporting decreased connection to others (62.3%), increased fatigue (63.9%), and decreased time spent communicating per individual (43.8%) (refer to Fig. 7). Impacts on communication quality in the workplace, how workers feel about communication, and how much time they spend communicating have implications for worker performance, worker engagement, and workplace health and safety. When public health regulations recommend or mandate the use of face masks, businesses and organisations must consider the impacts on their workers and, where possible, make allowances and adjustments for these impacts.

The proportion of participants reporting influence of face mask use by other people was significantly higher when communicating in the community or workplace than when communicating with household members. This is likely to be due to household members being more familiar with, and accommodating of, each other’s communication needs and communication styles. They were also not required to observe physical distancing rules. Observing such rules may compound the negative influence of face mask use on communication as the speech signal intensity is reduced at increased distance from the speaker. Nevertheless, 40–60% of those who communicated with household members whilst away from their home environment reported that face mask use by other household members had an influence on the quality of their communication, their feelings related to that communication, the time spent communicating, and the activities they participated in together (Fig. 1). In Australia, 71.3% of people live with a partner and/or other family member (Australian Bureau of Statistics, 2020). The communication setting of communicating with household members when away from the home environment was included in the survey because within-family communication and joint family activities are important to a sense of family cohesiveness. Particularly for children and parents, communication when away from the home is important and performs many functions, including those related to family bonding, personal safety, learning, and socialisation. Therefore, it is an important social finding that face mask use by household members had widespread influence on communication with those household members, the majority of whom are likely to be family members.

Approximately half of the participants reported influence of face mask use by other people on their own quality-of-life (Fig. 1). Although approximately half of the present participants self-reported no hearing difficulties, many of them reported a negative influence of face mask use by other people on communication (refer to the example in Table 3). It is reasonable to suggest there may be similarities between the experiences of listeners with hearing loss and those who find communication more difficult when others are wearing face masks, given that a prime driver of this increased difficulty is reduced speech understanding, and given that substantial numbers of participants reported increased feelings of communication-related anxiety and fatigue, and decreased satisfaction and connection to others. Hearing loss has previously been shown to be associated with reduced quality-of life for adults (Chia et al., 2007), including in the physical domain (Ciorba et al., 2012). The increased listening effort expended by adults with hearing loss has been associated with increased fatigue (Alhanbali et al., 2017). The complex relationship between hearing loss, fatigue, activity levels, and wellbeing was well described by Holman et al. (2019). Hearing loss has also been associated with loneliness and social isolation (Bott & Saunders, 2021; Maharani et al., 2019), each of which have been clearly shown to be connected to physical and mental health (Morina et al., 2021). Therefore, it is not surprising that participants who found it more difficult to communicate when other people were wearing face masks, even if they reported no hearing difficulties, would report influence of face mask use on their quality of life. Of course, as indicated by the odds ratios reported in Table 2 (OR 1.67–2.76), there was also increased likelihood of reporting influence for participants with increasing degree of self-reported hearing difficulties. The finding that, for some participants, face mask use by other people resulted in decreased connection to others and increased loneliness is particularly concerning. This is because other COVID-19 related public health orders which restricted person-to-person contact (stay-at-home orders, travel limitations, and physical distancing rules) have been shown to be associated with increased loneliness (Luchetti et al., 2020; McKenna-Plumley et al., , 2021). Thus, face mask use rules may compound the risk and/or degree of social isolation and loneliness experienced in the general community when combined with other public health orders during a pandemic. The same may be the case for other aspects of quality-of-life, such as worry and physical health.

### Study limitations

It is important to identify some methodological limitations. Firstly, the participant group was self-selected and therefore may not be truly representative of the broader population. For example, many more females than males completed the survey. Moreover, individuals who were having more difficulty communicating when other people wore face masks, or those who held strong views about mask-wearing, may have been more likely to participate. Those having less difficulty communicating may be under-represented. Secondly, the use of self-report to identify hearing difficulties may have underestimated the proportion of participants with hearing difficulties. This would not, however, have changed the overall proportion of individuals reporting influence from face mask use by other people. Thirdly, some participants may have over-reported influence due to conflation of the influence of face mask use by other people and other pandemic-related influences on their communication. To minimise this, participants were requested to specifically consider the influence of other people wearing face masks, rather than limitations (e.g. on allowed activities) and broader impacts (e.g. generally feeling more stressed) resulting from the pandemic. In addition, as noted above, the infection and death rate due to COVID-19 was relatively low in Australia prior to and during the data collection period.

## Conclusion

In the Australian state of Victoria, face mask use was mandatory in all settings outside the home for over 4 months in the second half of 2020. To varying degrees, face mask use by other people negatively influenced communication quality, feelings related to communication, and time spent communicating, with secondary effects indicated by reduced activity participation and, for a smaller proportion of participants, reduced quality-of-life. Greater influence of face mask use occurred for communication with community members in general, although communication with household members was also impacted. Influence of face mask use by other people was also strong in the workplace, affecting all aspects of communication and increasing negative emotions and fatigue. Female gender and increased degree of self-reported hearing difficulties were significantly associated with greater influence of face mask use by other people on a range of outcome variables; however, many participants reporting no hearing difficulties also reported influence due to face mask use. The wide-ranging influences of face mask use by other people have implications for physical health and mental health, including social connectedness, and for the economy. Workplace influences are potentially relevant to employee retention, productivity, and workplace health and safety. Although face masks remain an important public health measure, their negative impacts must be considered when formulating public health and workplace policies. When face masks are required, appropriate measures, such as educational campaigns to improve communication when face masks are in use, should also be put in place to reduce the impacts identified here. In particular, the experiences of women and those with self-reported hearing difficulties must be considered when designing and implementing interventions to improve communication.

## Supplementary Information

**Additional file 1**. List of survey items and response options.

## Data Availability

The list of survey items and responses is included as Additional file 1: Digital Content.
