# Supplementary material for: Effects of widespread community use of face masks on communication, participation, and quality of life in Australia during the COVID-19 pandemic
Source: Cogn Res Princ Implic. 2022 Oct 1;7:88. doi: 10.1186/s41235-022-00436-z (PMC9526537; doi:10.1186/s41235-022-00436-z)
Supplement: Supplementary file 1 — Additional file 1. List of survey items and response options. [file 41235_2022_436_MOESM1_ESM.docx]

Supplemental Digital Content 1: Survey Questions

In the past two weeks, how often did you leave your home for activities in the community, such as exercising, shopping or appointments (this does not include traveling to work or performing work-related activities)?

- Not at all
- Rarely (0 to 2 per week)
- Occasionally (3 to 5 times per week)
- Often (1 to 2 times per day)
- Very often (3 or more times per day)

In the past two weeks, thinking about the occasions when you left your home for activities in the community, how often did you communicate with people wearing face masks?

- Not at all
- Rarely (0 to 1 time per occasion when I left my home)
- Occasionally (2 to 4 times per occasion when I left my home)
- Often (5 to 10 times per occasion when I left my home)
- Very often (>10 times per occasion when I left my home)

*We are interested to know if the wearing of face masks by other people has had an impact on your communication, on how you feel about communication, or on your activities.* *The next questions relate to your communication with people wearing face masks and your activities when you are out in the community.*

*This question is about the* ***quality*** *of your communication with other people when you are out in the community. When thinking about the quality of communication, you might consider, amongst other things, how easy or hard the communication is, how complex the communication is, and how interrupted the communication is by the need for repeats or clarification.*

Has the wearing of face masks by other people influenced the quality of your communication with people in the community?

- No, not at all influential
- Somewhat influential
- Very influential

*In particular, how has the wearing of face masks by other people influenced the quality of your communication with people in the community?*

Communication has been

- easier.
- the same.
- harder.

I have needed

- less repetition.
- the same amount of repetition.
- more repetition.

I have needed

- less clarification.
- the same amount of clarification.
- more clarification.

I have been able to

- understand more.
- understand the same amount.
- understand less.

*This question is about* ***how you feel*** *when communicating with people out in the community. When thinking about how you feel, you might consider, amongst other things, your levels of fatigue, stress, anxiety, irritation, frustration, or enjoyment.*

Has the wearing of face masks by other people influenced your feelings related to your communication with people in the community?

- No, not at all influential
- Somewhat influential
- Very influential

*In particular, how has the wearing of face masks by other people influenced the quality of your communication with people in the community?*

I have felt

- less fatigued.
- the same.
- more fatigued.

I have felt

- less stressed.
- the same.
- more stressed.

I have felt

- less irritated.
- the same.
- more irritated.

I have felt

- less anxious.
- the same.
- more anxious.

I have felt

- less frustrated.
- the same.
- more frustrated.

I have experienced

- more enjoyment.
- the same amount of enjoyment.
- less enjoyment.

I have felt

- more satisfied.
- the same.
- less satisfied.

I have felt

- more connected to other people.
- the same.
- less connected to other people.

*This question is about the activities you engage in out in the community; these are the things you do when you leave your home. When thinking about your activities, consider activities you may* ***want*** *to do (e.g., talk to your neighbour on the street) and activities you may* ***need*** *to do (e.g., buy medicine from the pharmacy or chemist).*

Has the wearing of face masks by other people influenced what you do in terms of the activities you engage in out in the community? Remember we are interested in the influence of face masks, **not** other factors (such as fear of Covid-19 or restrictions which limit where you can go).

- No, not at all influential
- Somewhat influential
- Very influential

Do you do more or less of particular activities in the community because other people are wearing face masks? Remember we are interested in the influence of face masks, **not** other factors (such as fear of Covid-19 or restrictions which limit where you can go).

|  | More | The same | Less | Not applicable (e.g. not allowed; I don’t do this anyway) |
| --- | --- | --- | --- | --- |
| Shopping for medicines |  |  |  |  |
| Shopping for other necessary items (food, cleaning products, toiletries etc.) |  |  |  |  |
| Shopping for non-essential items (e.g., books, gifts) |  |  |  |  |
| Buying take-away food or drinks |  |  |  |  |
| Sitting down to eat at a café or restaurant |  |  |  |  |
| Talking with people I meet by chance |  |  |  |  |
| Meeting up with friends |  |  |  |  |
| Attending appointments with my doctor |  |  |  |  |
| Attending non-medical health-care appointments (e.g., physiotherapist) |  |  |  |  |
| Attending non-health related appointments (e.g. hairdresser) |  |  |  |  |
| Going to service locations (e.g. bank or post office) |  |  |  |  |
| Exercising |  |  |  |  |

Have any other of your activities out in the community been affected?

- Yes (please explain) ________________________________________________
- No

Has the wearing of face masks by other people influenced **how much time** you spend communicating with people in the community? Think about the number of people you communicate with and for how long you communicate with them.

- No, not at all influential
- Somewhat influential
- Very influential

Do you communicate with more people or fewer people in the community?

- More people
- The same number of people
- Fewer people

Do you communicate with individual people in the community for more time or less time?

- More time
- The same amount of time
- Less time

In the past two weeks, for how many days did you work at a usual workplace (e.g. in an office as a receptionist or in a school as a teacher), or engage in usual work-related activities outside the home (e.g. as a plumber or bus driver)? Your response should not include days working from home.

- None
- 1 to 4 days (i.e., average of 2 days per week) 5 to 7 days
- 8 to 10 days (i.e., almost full-time or full-time each week)

In the past two weeks, when at your workplace, or during work-related activities outside the home, how often did you communicate with people wearing face masks?

- Not at all
- Rarely (0 to 1 time per full day of work)
- Occasionally (2 to 4 times per full day of work)
- Often (5 to 10 times per full day of work)
- Very often (>10 times per full day of work)

| *This question is about the* ***quality*** *of your communication with other people when you are in the workplace or engaged in work-related activities outside the home. When thinking about the quality of communication, you might consider, amongst other things, how easy or hard the communication is, how complex the communication is, and how interrupted the communication is by the need for repeats or clarification.* |
| --- |

Has the wearing of face masks by members of your household influenced the quality of your communication with people at work?

- No, not at all influential
- Somewhat influential
- Very influential

| *In particular, how has the wearing of face masks by members of your household influenced the quality of your communication?* |
| --- |

Communication has been

- easier.
- the same.
- harder.

I have needed

- less repetition.
- the same amount of repetition.
- more repetition.

I have needed

- less clarification.
- the same amount of clarification.
- more clarification.

I have been able to

- understand more.
- understand the same amount.
- understand less.

| *This question is about* ***how you feel*** *when communicating with people in the workplace or engaged in work-related activities outside the home. When thinking about how you feel, you might consider, amongst other things, your levels of fatigue, stress, anxiety, irritation, frustration, or enjoyment.* |
| --- |

Has the wearing of face masks by other people influenced your feelings related to your communication with people at work?

- No, not at all influential
- Somewhat influential
- Very influential

| *In particular, how has the wearing of face masks by members of your household influenced the quality of your communication with them?* |
| --- |

I have felt

- less fatigued.
- the same.
- more fatigued.

I have felt

- less stressed.
- the same.
- more stressed.

I have felt

- less irritated.
- the same.
- more irritated.

I have felt

- less anxious.
- the same.
- more anxious.

I have felt

- less frustrated.
- the same.
- more frustrated.

I have experienced

- more enjoyment.
- the same amount of enjoyment.
- less enjoyment.

I have felt

- more satisfied.
- the same.
- less satisfied.

I have felt

- more connected to other people.
- the same.
- less connected to other people.

| *This question is about the activities you engage in when in the workplace or engaging in work-related activities outside the home. When thinking about your activities, consider activities you may* ***want*** *to do (e.g., talk to your colleague in the lunch room) and activities you may* ***need*** *to do (e.g., complete the job you are employed to do).* |
| --- |

Has the wearing of face masks by other people influenced the activities you engage in when at work? Remember we are interested in the influence of face masks, not other factors (such as fear of Covid-19 or restrictions which limit where you can go).

- No, not at all influential
- Somewhat influential
- Very influential

What activities do you engage in **more** at work due to other people wearing face masks? (optional)

What activities do you engage in **less** at work due to other people wearing face masks? (optional)

Has the wearing of face masks by other people influenced how much time you spend communicating with people at work? Think about the number of people you communicate with and for how long you communicate with them.

- No, not at all influential
- Somewhat influential
- Very influential

Do you communicate with more people or fewer people at work?

- More people
- The same number of people
- Fewer people

Do you communicate with individual people at work for more time or less time?

- More time
- The same amount of time
- Less time

Are there other people currently living with you in your household?

- No
- Yes

How many people are currently living with you in your household?

- Number of children ________________________________________________
- Number of adults ________________________________________________

In the past two weeks, how often did you leave your home for activities with someone from your household who was wearing a face mask?

- Not at all
- Rarely (0 to 2 per week)
- Occasionally (3 to 5 times per week)
- Often (1 to 2 times per day)
- Very often (3 or more times per day)

*This question is about the* ***quality*** *of your communication with members of your household when you are outside of your home together. When thinking about the quality of communication, you might consider, amongst other things, how easy or hard the communication is, how complex the communication is, and how interrupted the communication is by the need for repeats or clarification.*

Has the wearing of face masks by members of your household influenced the quality of your communication when you are outside of your home together?

- No, not at all influential
- Somewhat influential
- Very influential

*In particular, how has the wearing of face masks by members of your household influenced the quality of your communication?*

Communication has been

- easier.
- the same.
- harder.

I have needed

- less repetition.
- the same amount of repetition.
- more repetition.

I have needed

- less clarification.
- the same amount of clarification.
- more clarification.

I have been able to

- understand more.
- understand the same amount.
- understand less.

*This question is about* ***how you feel*** *when communicating with members of your household when outside of your home together. When thinking about how you feel, you might consider, amongst other things, your levels of fatigue, stress, anxiety, irritation, frustration, or enjoyment.*

Has the wearing of face masks by members of your household influenced your feelings related to your communication with them?

- No, not at all influential
- Somewhat influential
- Very influential

*In particular, how has the wearing of face masks by members of your household influenced the quality of your communication with them?*

I have felt

- less fatigued.
- the same.
- more fatigued.

I have felt

- less stressed.
- the same.
- more stressed.

I have felt

- less irritated.
- the same.
- more irritated.

I have felt

- less anxious.
- the same.
- more anxious.

I have felt

- less frustrated.
- the same.
- more frustrated.

I have experienced

- more enjoyment.
- the same amount of enjoyment.
- less enjoyment.

I have felt

- more satisfied.
- the same.
- less satisfied.

I have felt

- more connected to other people.
- the same.
- less connected to other people.

*This question is about the activities you engage in with members of your household when you are outside of your home together. When thinking about your activities, consider activities you may* ***want*** *to do (e.g. have conversation when out for a walk) and activities you may* ***need*** *to do (e.g. pick up your children from school).*

Has the wearing of face masks by members of your household influenced what you do in terms of the activities you engage in when outside of your home together? Remember we are interested in the influence of face masks, **not** other factors (such as fear of Covid-19 or restrictions which limit where you can go).

- No, not at all influential
- Somewhat influential
- Very influential

What activities do you perform **more** due to members of your household wearing masks when you are outside of your home together? (optional)

________________________________________________________________

What activities do you perform **less** due to members of your household wearing masks when you are outside of your home together? (optional)

________________________________________________________________

Has the wearing of face masks by members of your household influenced **how much time** you spend communicating with them when you are outside of your home together? Think about the number of people you communicate with and for how long you communicate with them.

- No, not at all influential
- Somewhat influential
- Very influential

Do you communicate with more or fewer members of your household when you are outside of your home together?

- More members of my household
- The same number of members from my household
- Fewer members of my household

Do you communicate with individual members of your household for more time or less time when you are outside of your home together?

- More time
- The same amount of time
- Less time

*This question relates to face mask use by* ***anyone else*** *(people in the community or workplace, or members of your household).*

Has the wearing of face masks by other people influenced your quality of life (e.g. happiness, loneliness, sleep) because of the impact of face masks on communication?

- No, not at all influential
- Somewhat influential
- Very influential

*In particular, how has the wearing of face masks by other people influenced your quality of life?*

I am

- happier.
- the same.
- less happy.

I

- worry less.
- worry the same amount.
- worry more.

I am

- less lonely.
- the same.
- more lonely.

My physical health is

- better.
- the same.
- worse.

My sleep is

- better.
- the same.
- worse.

Other (please specify):

________________________________________________________________
